# Prevalence and molecular characterizations of *Staphylococcus aureus* nasal colonization among patients in pediatric intensive care units in Taiwan

**DOI:** 10.1186/s13756-020-0700-6

**Published:** 2020-02-27

**Authors:** Yu-Hsin Chen, Kuan-Ying A. Huang, Yi-Chuan Huang, Hsin Chi, Chun-Yi Lu, Luan-Yin Chang, Yu-Huai Ho, Chia-Yu Chi, Ching-Chuan Liu, Li-Min Huang, Tien Yu Owen Yang, Yhu-Chering Huang

**Affiliations:** 1 Division of Pediatric Infectious Diseases, Department of Pediatrics, Chang Gung Memorial Hospital at Linkou, and College of Medicine, Chang Gung University, Taoyuan, Taiwan; 2 Research Center for Emerging Viral Infections, College of Medicine, Chang Gung University, Taoyuan, Taiwan; 3 Taiwan Pediatric Infectious Diseases Alliance, Taipei, Taiwan; 4 Division of Infectious Diseases, Department of Pediatrics, Kaohsiung Chang Gung Memorial Hospital and Chang Gung University College of Medicine, Kaohsiung, Taiwan; 5 Department of Pediatrics, Mackay Memorial Hospital, Taipei, Taiwan; 6 Department of Pediatrics, National Taiwan University Hospital, College of Medicine, National Taiwan University, Taipei, Taiwan; 7 Department of Internal Medicine, Buddhist Tzu Chi General Hospital, Hualien, Taiwan; 8 Department of Pediatrics, National Cheng-Kung University Hospital, Tainan, Taiwan; 9 Ph.D. Program in Environmental and Occupational Medicine, Kaohsiung Medical University, Kaohsiung, Taiwan; 10 National Institute of Infectious Diseases and Vaccinology, National Health Research Institutes, Miaoli County, Taiwan; 11 Nuffield Department of Population Health, University of Oxford, Oxford, UK

**Keywords:** *Staphylococcus aureus*, Nasal colonization, Prevalence, Molecular typing, Pediatric intensive care unit

## Abstract

**Background:**

Nasal colonization of *Staphylococcus aureus* is a risk factor for the pathogen transmission and the development of infections. Limited information is available on the prevalence and molecular characteristics of *S. aureus* colonization in pediatric intensive care unit (ICU) patients.

**Methods:**

A cross-sectional, island-wide study was conducted in 2011. Nasal swabs were collected from pediatric ICU patients at six tertiary hospitals in Taiwan.

**Results:**

Of 114 patients enrolled in total, nasal colonization of *S. arueus* was detected in 30 (26.3%) of them, among whom 20 (17.5%) with methicillin-resistant *S. arueus* (MRSA). The ST59/SCC*mec* IV and V clones were most common and accounted for 45% of MRSA isolates, followed by ST239/SCC*mec* III (25%) and ST45/SCC*mec* IV (20%) clones. Three ST59 MRSA isolates carried the Panton-Valentine Leukocidin genes.

**Conclusions:**

The results indicated a high prevalence of *S. arueus* and MRSA nasal colonization among pediatric ICU patients in Taiwan. Identification of epidemic clones warrants the implement of infection control measures to reduce colonization and prevent the dissemination of MRSA in hospitals.

## Background

*Staphylococcus aureus* is a primary cause for hospital- and community-associated bacterial infections worldwide [1]. It is estimated that over one million *S. aureus* skin and soft tissue infections occur in the United States every year, potentially leading to around 100,000 bacteremia and 20,000 deaths [2]. Among clinical *S. aureus* isolates, methicillin-resistant *S. aureus* (MRSA) has emerged as a widespread pathogen in both the community and hospital settings [1]. In Taiwan, the MRSA has accounted for as much as 50–80% of all *S. aureus* isolates since 1990s [3, 4]. Moreover, the MRSA bacteremia is associated with an increased risk of mortality compared with the methicillin-susceptible *S. aureus* (MSSA) bacteremia [5].

Colonization with *S. aureus* is a risk factor for the development of clinical *S. aureus* infection [6, 7]. A large-scale prospective study showed that nosocomial *S. aureus* bacteremia is three times more frequent in nasal *S. aureus* carrier than in non-carriers, which indicates colonized patients are probably the main source of *S. aureus* in hospitals [7]. Another study further showed that MRSA-colonized patients are more likely to develop a MRSA invasive infection, compared with MSSA-colonized or non-colonized patients, even after adjusting for patient-specific risk factors such as comorbidities [8]. Over recent years, the continuing increase in the prevalence of nosocomial infections involving multidrug-resistant *S. aureus* has represented a critical and growing threat to human health [1, 2]. Another concerning issue is the rapid emergence and clonal spread of community-associated strains of MRSA, which often produce virulent exotoxins, i.e. Panton-Valentine leukocidin [9–11]. The identification of high-risk groups for carrying *S. aureus* and MRSA and the delineation of the frequency and molecular epidemiology of colonizing strains will provide valuable information for formulating effective measures in controlling the spread of *S. aureus* and MRSA in the community and hospital.

The anterior nares are the main reservoir of *S. aureus* colonization, but *S. aureus* can also be found in the oral cavity, perineum, axilla and on the skin [12]. In humans, nasal colonization of *S. aureus* may begin within the first days of life [13, 14]. In the general population, nasal *S. aureus* and MRSA carriages are more common in children than those in young adults and elders, but the carriage prevalence varies geographically [15–17]. In the hospital, the prevalence of *S. aureus* colonization was investigated among non-surgical patients, patients on hemodialysis or HIV-infected patients [7, 18, 19]. However, limited data is available for the prevalence of *S. aureus* and MRSA colonization in intensive care unit (ICU) patients [20].

Here, we conducted an island-wide survey to investigate the prevalence of nasal *S. aureus* colonization among pediatric ICU patients, to delineate molecular characteristics and antimicrobial resistance profiles of MRSA, and to determine the demographic and clinical characteristics associated with the MRSA colonization among six participating tertiary hospitals in Taiwan.

## Methods

### Study design and sample collection

The study is a cross-sectional study involving six tertiary hospitals in Taiwan. Patients who were admitted to the pediatric ICUs of the six hospitals on two designated dates, i.e., October 11 (first survey) and December 12 (second survey), 2011, were eligible for and all were enrolled in this study. The six participating hospitals included Taipei Mackay Memorial Hospital (MM), Linkou Chang Gung Memorial Hospital (LC), National Taiwan University Hospital (T), Kaohsiung Chang Gung Memorial Hospital (KC), National Cheng Kung University Hospital (CK) and Hualien Tzu-Chi General Hospital (TC). All six participating hospitals are tertiary medical centers, of which MM, LC and T are located in northern Taiwan, KC and CK are located in southern Taiwan and TC is located in eastern Taiwan. In total, there were 85 pediatric ICU beds (12 beds in MM, 20 beds in LC, 20 beds in T, 20 beds in KC, 8 beds in CK and 5 beds in TC). The pediatric ICU bed occupancy rate for each participating hospital varies with the number of visited patients and the season.

One nasal swab was obtained from each patient and sent to the central laboratory at National Health Research Institutes for the detection of *S. aureus* by standard methods [21]. Briefly, the swab sample was inoculated onto trypticase soy agar with 5% sheep blood plates by the streak-plating method. After incubation at 37 °C overnight, the colony of suspected *S. aureus* based on the hemolysis pattern and other macroscopic appearances, if any, was further inoculated on another 5% sheep blood plates. A coagulase test was performed to ensure the identification of *S. aureus*. A cefoxitin disk diffusion test was used to distinguish MRSA from MSSA in accordance with the recommendations of the CLSI document M100-S20 [22]. All the *S. aureus* isolates were stored for further molecular characterization.

### Molecular characterization

Pulsed-field gel electrophoresis (PFGE) with *Sma*I digestion was performed as previously described [23]. The genotypes which were designated were in line with a previous survey and were listed in alphabetical order and we designated the newly identified genotype consecutively [23]. Four or more band differences between two isolates defined different genotypes. Multilocus sequence typing (MLST) was performed by analyzing the DNA sequences of seven housekeeping genes with known alleles at each locus through the MLST website (http://www.mlst.net) [24].

The staphylococcal cassette chromosome *mec* (SCC*mec*) typing was performed by multiplex polymerase chain reaction as previously described [25]. The presence of virulence genes, including enterotoxin A, B, C, Panton-Valentine leukocidin (PVL), toxic shock syndrome toxin-1 (TSST-1), exfoliative toxin A (Eta), and fibronectin-binding protein A (FnbA), was examined by polymerase chain reaction analysis.

### Antimicrobial susceptibility test

Susceptibility to the antibiotics, including clindamycin, erythromycin, doxycycline, tetracycline, gentamicin, levofloxacin, trimethoprim/sulfamethoxazole, rifampin and vancomycin, was performed on Mueller-Hinton agar plates by modified Kirby-Bauer disc diffusion method. The disc diffusion technique and zone interpretation of each antimicrobial agent were performed in accordance with the CLSI guideline [22]. *Staphylococcus aureus* ATCC-25923 was used as a standard control strain.

### Statistics

The statistical analysis was performed with the SPSS software (SPSS, Chicago, USA) and SAS 9.3 software (SAS Institute, Cary, USA). We calculated the colonization rates in all patients enrolled, by hospitals and by 2 surveys, and by age, sex, and clinical characteristics, including diagnosis of skin and soft tissue infections at admission, use of nasogastric or nasoduodenal tube, prolonged stay in the PICU (> 3 weeks), history of hospitalization in the past year, history of MRSA colonization or infection in the past year, and antibiotics use during the current hospitalization. Chi-squared tests or Fisher’s exact tests, where appropriate, were used to compare differences in colonization rates among subgroups by characteristics. A *P* value < 0.05 was considered significant in these comparisons. Study quality was assessed using the Strengthening the Reporting of Observational Studies in Epidemiology (STROBE) checklist for cross-sectional studies (Additional file 1).

## Results

### The prevalence of *S. aureus* colonization

A total of 114 patients were admitted to pediatric ICUs at two time points (56 in October 10th and 58 in December 12th, 2011, respectively) and all were enrolled in the study. Table 1 showed the number of samples across six hospitals. Of the 114 patients, 30 (26.3%) harbored *S. aureus* and MSSA and MRSA were identified from 11 (9.6%) and 20 (17.5%) patients, respectively. We noted that one patient carried both MSSA and MRSA. Another patient provided samples at two time points and the MRSA was identified from both samples.

**Table 1:** Numbers of patients with nasal colonization of methicillin-sensitive *S. aureus* (MSSA) and of methicillin-resistant *S. aureus* (MRSA) in pediatric intensive care units by six tertiary hospitals

Site	First survey^a^					Second survey^a^					Total				
	*n*	MSSA		MRSA		*n*	MSSA		MRSA		*n*	MSSA		MRSA	
		*n*	(%)	*n*	(%)		*n*	(%)	*n*	(%)		*n*	(%)	*n*	(%)
LC	16	0	(0.0)	4	(25.0)	17^c^	2	(11.8)	5	(29.4)	33	2	(6.1)	9	(27.3)
KC	15	1	(6.7)	1	(6.7)	12	1	(8.3)	4	(33.3)	27	2	(7.4)	5	(18.5)
T	12	1^b^	(8.3)	2^b^	(16.7)	10	1	(10.0)	2	(20.0)	22	2	(9.1)	4	(18.2)
CK	5	1	(20.0)	0	(0.0)	7	2	(28.6)	1	(14.3)	12	3	(25.0)	1	(8.3)
MM	9	0	(0.0)	0	(0.0)	10	2	(20.0)	1	(10.0)	19	2	(10.5)	1	(5.3)
TC	1	0	(0.0)	0	(0.0)	0	0	(0.0)	0	(0.0)	1	0	(0.0)	0	(0.0)
Total	58	3	(5.2)	7	(12.1)	56	8	(14.3)	13	(23.2)	114	11	(9.6)	20	(17.5)

^a^ *LC* Linkou Chang Gung Memorial Hospital, *KC* Kaohsiung Chang Gung Memorial Hospital, *T* National Taiwan University Hospital, *CK* National Cheng Kung University Hospital, *MM* Taipei Mackay Memorial Hospital, *TC* Hualien Tzu-Chi General Hospital, first survey performed on October 11th, 2011 and second survey performed on December 12th, 2011.

^b^ One patient had both MSSA and MRSA colonization

^c^ One patient was repeatedly enrolled in the first and second surveys

The MRSA colonization rate ranged from 0 to 27.3% in the six hospitals (P for between-hospital difference = 0.44, Chi-square test). The colonization rate of MRSA was 12.1% (7/58) in October and 23.2% (13/56) in December, 2011 (P for between-survey difference = 0.14, Fisher’s exact test).

Table 2 showed that the MRSA colonization in subgroups by age, sex and clinical characteristics. The numbers in subgroups were small and therefore most statistical tests were underpowered. Nevertheless, with weak statistical significance the colonization rates were found to vary with age (P for rate difference by age = 0.02, Chi-Square test), where the rate among the youngest children (< 1 year old) was found lower than children of older age groups (3.0, 28.9, and 25.0% for < 1 years, 1–6 years, and 7–12 years old, respectively). Despite some rate differences by other characteristics, these differences were not statistically significant by sex, diagnosis of skin and soft tissue infections at admission, use of nasogastric or nasoduodenal tube, prolonged length of stay, history of hospitalization in the past one year, history of MRSA colonization or infection in the past one year, and the antibiotics usage during the hospitalization (P for difference > 0.05 in all comparisons).

**Table 2:** MRSA nasal colonization rates among subgroups by age, sex, and clinical characteristics

Demographic and clinical characteristics	MRSA colonization rate	*P* value
Age, years		
< 1	1/33 (3.0)	
1–6	13/45 (28.9)	
7–12	4/16 (25.0)	
≧13	2/20 (10.0)	0.02
Sex		
Male	11/68 (16.2)	
Female	9/46 (19.6)	0.80
Diagnosis of SSTIs at admission		
Yes	1/2 (50.0)	
No	19/112 (17.0)	0.32
Nasogastric or nasoduodenal tube		
Yes	12/58 (20.7)	
No	8/56 (14.3)	0.06
Length of stay > 3 weeks		
Yes	2/33 (6.1)	
No	18/81 (22.2)	0.06
History of hospitalization in the past 1 year		
Yes	14/76 (18.4)	
No	6/38 (15.8)	0.80
History of MRSA colonization or infection in the past 1 year		
Yes	4/12 (33.3)	
No	16/102 (15.7)	0.22
Antibiotics used in the current hospitalization		
Number of agents		
0	2/16 (12.5)	
1–3	14/68 (20.6)	
≧4	4/30 (13.3)	0.58
Class of agents		
Penicillin	11/47 (23.4)	
Cephalosporin	8/63 (12.7)	
Carbapenem	3/23 (13.0)	
Glycopeptide	7/43 (16.3)	
Quinolone	1/10 (10.0)	0.59

*MRSA* methicillin-resistant *Staphylococcus aureus*, *SSTI* skin and soft tissue infections

### Molecular characteristics and antimicrobial susceptibility profiles of MRSA isolates

**Fig. 1:**
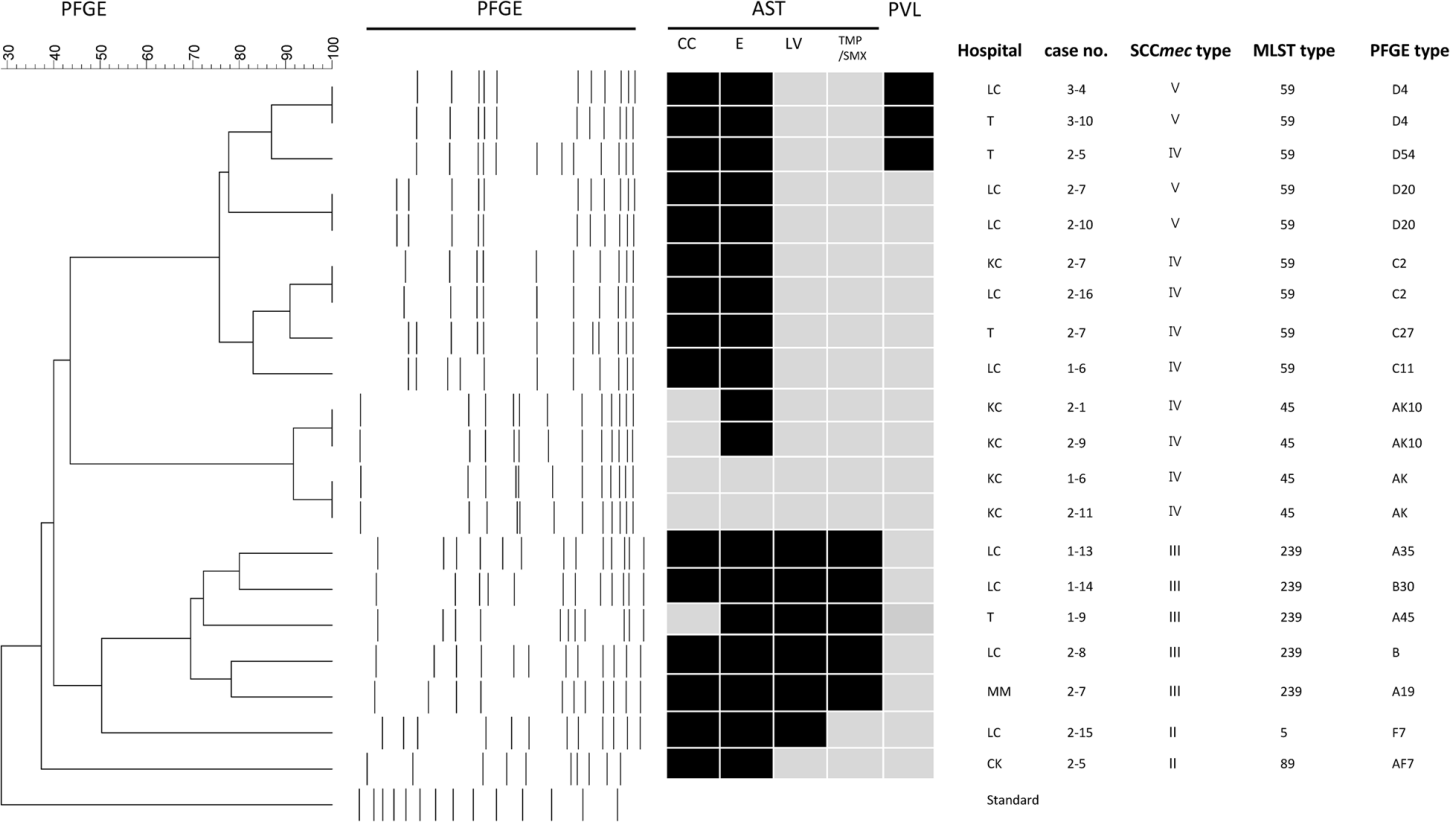
The molecular characteristics of MRSA isolates. For the antimicrobial susceptibility test (AST), the black and grey bars represent resistance and susceptibility to the antibiotic, respectively. Abbreviations: PFGE, pulsed-field gel electrophoresis; CC, clindamycin; E, erythromycin; LV, levofloxacin; TMP/SMX, trimethoprim/sulfamethoxazole; PVL, Panton-Valentine leucocidin; LC, Linkou Chang Gung Memorial Hospital in northern Taiwan; T, National Taiwan University Hospital in northern Taiwan; KC, Kaohsiung Chang Gung Memorial Hospital in southern Taiwan; MM, Taipei Mackay Memorial Hospital in northern Taiwan; CK, National Cheng Kung University Hospital in southern Taiwan; MLST, multilocus sequence typing

The 20 MRSA isolates were classified into five distinct clonal lineages by MLST, of which ST59 (9/20, 45%) being most common, followed by ST239 (5/20, 25%) and ST45 (4/20, 20%) clones (Table 3 and Fig. 1).

**Table 3:** Molecular characteristics and antimicrobial susceptibility rates (%) among the 20 MRSA colonizing isolates by staphylococcal cassette chromosome *mec* (SCC*mec*) types

	SCC*mec* type			
	II (*n* = 2)	III (*n* = 5)	IV (*n* = 9)	V (*n* = 4)
Molecular characteristics				
MLST	5, 89	239	45 (4), 59 (5)	59
PFGE	F, AF	A (3), B (2)	C (4), D (1), AK (4)	D
Antimicrobial susceptibility				
Doxycycline	100	80	100	100
Tetracycline	100	0	78	25
Gentamicin	50	0	78	100
Clindamycin	0	20	44	0
Erythromycin	0	0	22	0
Levofloxacin	50	0	100	100
TMP/SMX	100	0	100	100
Rifampin	100	100	100	100
Vancomycin	100	100	100	100

*MLST* multilocus sequence type, *PFGE* pulsed-fielded gel electrophoresis, *TMP/SMX* trimethoprim/sulfamethoxazole

As shown in Table 3 and Fig. 1, PFGE was able to divide the most prevalent ST59 isolates into two major pulsotypes (type C and D), the majority of type D isolates carried SCC*mec* V and all type C isolates carried SCC*mec* IV. The ST45 isolates shared similar PFGE pattern, designated as type AK, and all carried SCC*mec* IV. ST239 isolates were shared by two pulsotypes, namely type A and B, but all ST239 isolates carried SCC*mec* III. While ST59 and ST239 clones were detected from pediatric ICU patients at various hospitals, the ST45 clone was predominantly found from patients of KC hospital located in southern Taiwan.

Three of 20 MRSA isolates were PVL-positive, of which two were ST59/SCC*mec* V and the other was ST59/SCC*mec* IV isolate (Fig. 1). As shown in Table 4, the PVL genes and enterotoxin B genes were only detected in MRSA isolates, while none of MSSA isolates carried these genes.

**Table 4:** Virulence genes in *S. aureus* isolates

Toxin	Isolates with gene, n (%)		*P* value
	MRSA (*n* = 20)	MSSA (*n* = 11)	
Enterotoxin			
A	4 (20.0)	3 (27.2)	0.68
B	7 (35.0)	0 (0.0)	0.03
C	5 (25.0)	2 (18.2)	> 0.9
PVL	3 (15.0)	0 (0.0)	0.54
TSST-1	3 (15.0)	2 (18.2)	> 0.9
Eta	0 (0.0)	2 (18.2)	0.12
FnbA	20 (100.0)	11 (100.0)	> 0.9

*MRSA* methicillin-resistant *Staphylococcus aureus*, *MSSA* methicillin-susceptible *Staphylococcus aureus*, *PVL* Panton-Valentine leukocidin, *TSST-1* Toxic shock syndrome toxin-1, *Eta* exfoliative toxin A, *FnbA* Fibronectin-binding protein A, *NA* non-available

The 20 MRSA isolates showed variable rates of resistance to antibiotics (Table 3). The rates of resistance to doxycycline, tetracycline, gentamicin, clindamycin, erythromycin, levofloxacin and trimethoprim-sulphamethoxazole were 5, 50, 40, 75, 90, 30 and 25%, respectively. None of the isolates were resistant to rifampin and vancomycin.

Isolates of different clonal complexes exhibited distinct patterns of antibiotic susceptibilities (Table 3 and Fig. 1). The ST239 isolates were resistant to multiple antibiotics, including clindamycin, erythromycin, levofloxacin and trimethoprim-sulphamethoxazole. By contrast, ST59 and ST45 isolates were less resistant and both were susceptible to levofloxacin and trimethoprim-sulphamethoxazole. Besides, all ST45 isolates were susceptible to clindamycin and 50% of isolates were susceptible to erythromycin.

## Discussion

The island-wide survey of *S. aureus* colonization among patients in pediatric ICUs showed that the *S. aureus* and MRSA nasal colonization rates were 26.3 and 17.5% in Taiwan, respectively. The MRSA nasal colonization rate in this study was relatively higher than that in pediatric ICU patients in the USA (4.5–6.0%) [26, 27], Saudi Arabia (2.7%), or United Kingdom (1.6–2.9%) [28]. Firstly, the high colonization rate may be associated with the local MRSA epidemiology. In community-level studies, the rates of *S. aureus* and MRSA nasal colonization were as high as 22.0–30.1% and 7.8–17.6%, respectively, among healthy Taiwanese children in the 2000s [23, 29]. Recently, longitudinal surveys showed that around 40% of healthy Taiwanese children ever carried MRSA during the first 2 years of life [14, 30]. Secondly, although the transmission of *S. aureus* between healthcare workers, the environment, and patients is an infrequent occurrence, health-care workers are possible sources of MRSA transmission to patients [31]. Previous investigation has showed around 6% of healthcare workers carry MRSA in Taiwan [32]. Taken together, our data suggested that MRSA nasal colonization was common among pediatric patients in the hospital setting in Taiwan. Although the information about the development of subsequent *S. aureus* and MRSA infections among colonized patients is lacking in the study, previous study found that the relative risk for MRSA infections among pediatric patients who are colonized with MRSA on admission to ICUs are 24.2 compared with those who are not colonized [28]. Continuing surveillance in the MRSA carriage among pediatric ICU patients and a follow-up study to determine related clinical burden are warranted in Taiwan.

The clonal analysis in the study showed that 45% of MRSA nasal isolates belonged to a local community-associated MRSA linage, ST59, and carried either SCC*mec* IV or SCC*mec* V. The ST59/SCC*mec* V is the most prevalent clone in community-associated MRSA isolates in Taiwan and referred as Taiwan clone [4]. The ST59/SCC*mec* V clone often carries PVL genes, but the other clone, the ST59/SCC*mec* IV clone, is mostly PVL-negative (Fig. 1) [4, 33]. The emergence and clonal spread of ST59/SCC*mec* IV isolates in both the community and hospital have been reported in Taiwan and other regions since the early 2000s [10, 11, 33]. Recently, it was found that the ST59/SCC*mec* IV accounts for 50–60% of colonizing MRSA isolates in the community in Taiwan [23, 32]. Increasing evidence shows that the spread of community associated-MRSA would affect the colonization trends in the hospital and long-term care settings [26, 34, 35], which is further supported by the high detection of community associated-MRSA carriage among inpatients in the present study.

The ST239/SCC*mec* III clone was the second common MRSA clone in this survey. This clone is a worldwide-disseminated lineage of hospital-associated MRSA [36]. In Taiwan, the ST239 lineage appeared in the 1990s, and remained one of the dominant hospital-associated MRSA clones in 2010 [4]. A recent study demonstrated that the virulence factor *sas*X*/ses*I in the ST239 MRSA plays a key role in nasal colonization and the pathogenesis of severe infections [37]. It is suggested that hospital-associated MRSA may continuously colonize and contribute to a reservoir of MRSA infection in the hospital [38]. Similar to the findings of previous studies, this survey revealed that the ST239 clonal lineage contributes to one of major MRSA colonizing clones in hospitals and is resistant to multiple antibiotics such as erythromycin, gentamicin, sulfamethoxazole/trimethoprim, levofloxacin and tetracycline [32, 39].

A subset of MRSA colonizing isolates, characterized as the ST45/SCC*mec* IV clone, was identified from one hospital in southern Taiwan. In Taiwan, the ST45 clone was firstly identified in an MRSA outbreak in the respiratory ward in 2006 [40] and accounted for 50% of the MRSA colonizing isolates among nursing home residents and staffs in a recent study [41], indicating the transmission and spread of this MRSA clone in healthcare facilities. The ST45 clone was rarely reported in Asia before. However, in the past decade, the ST45 emerged and became prevalent in healthcare settings in Hong Kong and China [42]. The recent study found that the ST45 clone has replaced ST239 as the second leading MRSA nasal colonization strain among patients and healthcare workers in central Taiwan [32] and further molecular characterization and epidemiology should be conducted in the near future.

Several clinical factors may contribute to the MRSA colonization. Previous antimicrobial use has been associated with the MRSA colonization in hospitalized patients, particularly those in ICUs [43]. Other factors included the contact with a health-care facility (the length of stay, history of hospitalization) and personal medical background (diagnosis of skin and soft tissue infections, placement of nasal gastric tube) [43, 44]. Although we found no statistical evidence suggesting different rates of MRSA nasal colonization by these factors other than age, the case number was small and the statistical tests were underpowered. We noted that a majority of young pediatric patients (62% of patients aged 1–6 years) was colonized with the ST59 community-associated MRSA. Although it remains debatable whether frequent person-to-person contacts in the day care center lead to an increased risk of MRSA transmission in young children [45, 46], the spread of MRSA in the community among susceptible individuals may play a role and continues to be a serious issue.

Our results showed that nearly one of six patients in pediatric ICUs in Taiwan carries MRSA, among which local community and hospital clones are identified. These findings have implications for infection control of MRSA. Strict hand hygiene before and after patient contact is the primary measure in preventing and controlling the spread of MRSA [31, 47]. Environmental hygiene is also important to reduce the MRSA reservoirs and the transmission of MRSA in the clinical setting [31]. MRSA decolonization has been proposed as a potential strategy to reduce nosocomial spread of MRSA colonization and the subsequent risk of infection [31, 48]. A study of adult ICU patients supports universal decolonization for prevention of MRSA infections [49]. Evidence also shows targeted decolonization might reduce the risk of subsequent MRSA infection in colonized neonates [50, 51]. However, the study about the efficacy of MRSA decolonization in pediatric ICUs is limited and the optimal protocol for such patients is undetermined [52]. A study in the pediatric ICU showed a significant association between 2% chlorhexidine bathing and a decrease in Gram-positive bacteremia compared with standard bathing [53]. It is generally accepted that decolonization may be considered if a patient develops a recurrent MRSA infection despite the implementation of standard infection control measures; nevertheless, the establishment of decolonization policy with favorable outcomes among pediatric ICU patients would be the necessary next step in this effort. Active surveillance has been considered to detect asymptomatic carriers who could be a possible source of nosocomial MRSA transmission and thus infection control measures can be implemented early on [31, 52], but the routine screening for MRSA would be time-consuming and costly and may have practical considerations. At last, we suggest that pediatric patients and their caregivers should be informed of the MRSA-carriage status and further education about the significance of MRSA and the method to reduce transmission should be provided to the patient and their caregivers as well when the patient is discharged.

There are limitations of this study. Firstly, the study was conducted in 2011 and a new survey to update the prevalence and molecular characteristics of *S. aureus* nasal carriage in pediatric ICUs is important and should be conducted in the near future. Since the early 2010s, an estimated proportion from 28% to over 70% among clinical *S. aureus* isolates has been MRSA in hospitals in Asia [4]. In Taiwan, the *S. aureus* and MRSA continue to be the leading cause of skin and soft tissue infections and invasive bacterial infections in children [38, 54]. Recent reports show that ST59, ST239 and ST45 clones remain prevalent in clinical and colonizing MRSA isolates [32, 35], which is compatible with the findings in the present island-wide survey and indicates that our data is still relevant for the local epidemiology of MRSA in Taiwan today. Secondly, only one patient was admitted to the pediatric ICU and enrolled at the TC (Hualien Tzu Chi Hospital). The TC is the one and only medical center on the east of Taiwan where is a sparsely populated area compared to other areas of Taiwan. The low occupancy rate of the pediatric ICU may partially explain a small number of enrolled patients at the TC. Thirdly, nasal swab was used in the study for active surveillance of *S. aureus* colonization. Although nasal swab is the most common method, a combination of sampling from two and more sites, such as throat, groin or axilla, may improve the detection of MRSA colonization [55]. Fourthly, the MRSA carriage could be persistent or intermittent, where persons are colonized for a short time period. Nevertheless, the duration of MRSA nasal colonization among our pediatric ICU patients was unclear in this cross-sectional study.

## Conclusions

A high prevalence of MRSA and *S. aureus* colonization was observed in pediatric ICU patients. Identification of dominant MRSA clones in hospitals island-wide warrants an effective infection control measure to reduce nasal colonization and to prevent NRSA dissemination in the hospital setting.

## Supplementary information

**Additional file 1.** STROBE Statement—Checklist of items that should be included in reports of cross-sectional studies

## Data Availability

The datasets used and/or analyzed during the current study are available from the corresponding author on reasonable request.

## Abbreviations

CK: National Cheng Kung University Hospital
Eta: Exfoliative toxin A
FnbA: Fibronectin-binding protein A
ICU: Intensive care unit
KC: Kaohsiung Chang Gung Memorial Hospital
LC: Linkou Chang Gung Memorial Hospital
MLST: Multilocus sequence typing
MM: Taipei Mackay Memorial Hospital
MRSA: Methicillin-resistant *S. aureus*
MSSA: Methicillin-susceptible *S. aureus*
PFGE: Pulsed-field gel electrophoresis
PVL: Panton-Valentine leucocidin
SCC*mec*: Staphylococcal cassette chromosome *mec*
T: National Taiwan University Hospital
TC: Hualien Tzu-Chi Hospital
TSST-1: Toxic shock syndrome toxin-1
